# Moonlighting ERAP Aminopeptidases in Cancer: Beyond Antigen Processing

**DOI:** 10.3390/cells15171609

**Published:** 2026-09-04

**Authors:** Paula Gragera, Valentina Scaldaferri, Doriana Fruci

**Affiliations:** Ospedale Pediatrico Bambino Gesù IRCCS, 00146 Rome, Italy; paula.grageraalvarez@opbg.net (P.G.); valentina.scaldaferri@opbg.net (V.S.)

**Keywords:** ERAP1, ERAP2, antigen processing, cancer, cancer immunotherapy, targeted therapy, precision oncology

## Abstract

**Highlights:**

**What are the main findings?**

- ERAP1 and ERAP2 are key regulators of the MHC class I immunopeptidome that influence both adaptive and innate anti-tumor immune responses.
- Beyond antigen processing, ERAP aminopeptidases display moonlighting functions in inflammation, angiogenesis, ER stress, cell migration, and tumor-intrinsic signaling.

**What are the implications of the main findings?**

- Pharmacological targeting of ERAP enzymes, particularly ERAP1, represents a promising strategy to remodel tumor immunogenicity and overcome resistance to cancer immunotherapy.
- Understanding the canonical and non-canonical functions of ERAP aminopeptidases may support the development of next-generation precision immuno-oncology approaches.

**Abstract:**

Cancer immunotherapy has transformed the treatment of multiple malignancies; however, primary and acquired resistance remain major clinical challenges. Because effective immune recognition depends on the repertoire of peptides presented by major histocompatibility complex class I (MHC-I) molecules, increasing attention has focused on the antigen processing and presentation pathway as a therapeutic target to enhance tumor immunogenicity. Among its key regulators, the endoplasmic reticulum (ER) aminopeptidases ERAP1 and ERAP2 shape the MHC-I immunopeptidome by trimming peptide precursors before antigen presentation. Beyond this canonical function, accumulating evidence indicates that ERAP aminopeptidases are multifunctional proteins involved in inflammation, angiogenesis, ER stress responses, cell migration, and tumor-intrinsic signaling. These moonlighting activities suggest that ERAP enzymes influence cancer progression through both immune-dependent and immune-independent mechanisms. Recent advances in medicinal chemistry have enabled the development of selective ERAP1 inhibitors, leading to the first clinical evaluation of this therapeutic strategy and providing early clinical evidence that pharmacological modulation of antigen processing may complement existing immunotherapies. In this review, we summarize the multiple functions of ERAP aminopeptidases in cancer, discuss their role in regulating adaptive and innate immune responses, and highlight emerging therapeutic strategies and future challenges for exploiting ERAP-targeted interventions in precision immuno-oncology.

## 1. Introduction

Cancer immunotherapy is revolutionizing the treatment of several malignancies, demonstrating that durable tumor control can be achieved through the activation of the endogenous immune system [1,2]. Immune checkpoint inhibitors (ICIs), adoptive cell therapies, and therapeutic cancer vaccines have produced remarkable clinical benefits in a subset of patients, establishing anti-tumor immune recognition as a critical determinant of therapeutic success [2,3,4]. To enhance the efficacy of these approaches, current strategies increasingly explore the combination of ICIs with conventional neoadjuvant cytotoxic regimens to improve pathological responses and ultimately clinical outcomes [5]. Nevertheless, many patients either fail to respond or eventually develop acquired resistance, highlighting the need for strategies that can broaden and sustain the efficacy of current immunotherapies.

One of the major barriers to effective anti-tumor immunity is the ability of cancer cells to evade immune recognition [6,7,8,9]. The activity of cytotoxic CD8^+^ T cells and natural killer (NK) cells relies on the molecular visibility of tumor cells, which is largely determined by the repertoire of peptides presented by major histocompatibility complex class I (MHC-I) molecules on the cell surface [10]. This peptide repertoire is generated by the antigen processing and presentation (APP) pathway, which determines the identity of the antigens exposed to the immune system. By controlling which peptides are generated, selected, and displayed by MHC-I molecules, the APP machinery ultimately determines whether malignant cells are recognized and eliminated or escape immune surveillance [11].

Given its central role in shaping tumor immunogenicity, the APP pathway has emerged as an attractive therapeutic target. Manipulating antigen processing offers the opportunity to remodel the MHC-I immunopeptidome, enhance tumor visibility to immune effector cells, and ultimately improve the efficacy of existing cancer immunotherapies. Among the regulators of the MHC-I APP pathway, the endoplasmic reticulum (ER) aminopeptidases 1 and 2 (ERAP1 and ERAP2, respectively) have emerged as key determinants of antigen presentation. By shaping the repertoire of peptides displayed by MHC-I molecules, these enzymes influence the visibility of tumor cells to both adaptive and innate immune responses [12].

Beyond their established role in the APP pathway, ERAP aminopeptidases are increasingly recognized as multifunctional proteins with additional roles in inflammation, angiogenesis, and tumor biology, suggesting that their impact on cancer extends beyond immune surveillance. These functions, together with the possibility of pharmacologically modulating their activity, have positioned ERAP enzymes as emerging targets at the interface between antigen processing, tumor biology, and cancer immunotherapy.

In this review, we discuss the current understanding of ERAP aminopeptidases in cancer, focusing on their canonical role in shaping the tumor immunopeptidome, as well as their emerging non-canonical functions. We provide a cancer-focused perspective that integrates mechanistic insights into ERAP biology with the evolving clinical landscape of ERAP-targeted therapies, highlighting their therapeutic potential and prospects for clinical translation.

To provide an evidence-based and structured overview of this rapidly evolving field, we conducted a targeted literature search covering studies published through July 2026. The search was performed primarily using PubMed/MEDLINE, with specific search terms and combinations including “ERAP1”, “ERAP2”, “endoplasmic reticulum aminopeptidase”, “ARTS-1”, “antigen processing and presentation”, “MHC-I immunopeptidome”, “cancer immunotherapy” and “targeted therapy”. To capture seminal studies that might not be identified through keyword-based searches alone, the database search was complemented by recursive screening of references from key primary studies and authoritative reviews. In addition, relevant high-impact clinical developments and preliminary trial findings presented at recent international oncology meetings were considered. English-language primary research articles and peer-reviewed reviews were screened and evaluated, with particular emphasis on studies providing mechanistic insights or directly informing the role of ERAPs in anti-tumor immunity. By integrating evidence from molecular and biochemical studies, preclinical models, and emerging clinical data, this narrative review aims to provide a comprehensive framework for understanding the diverse biological functions of ERAPs and their potential as targets for precision cancer immunotherapy.

## 2. Canonical Role of ERAP Aminopeptidases in Antigen Processing and Presentation

The generation and presentation of peptides by MHC-I molecules is a tightly regulated process that defines the repertoire of antigens available for recognition by cytotoxic lymphocytes. Following proteasomal degradation of intracellular proteins, peptide precursors are transported into the ER through the transporter associated with antigen processing (TAP). Because many TAP-delivered peptides exceed the optimal length required for stable MHC-I binding, they undergo a final trimming step within the ER. This process is catalyzed by the zinc-dependent M1 aminopeptidases ERAP1 and ERAP2, which remove N-terminal residues from peptide precursors, typically 10 to 24 amino acids in length, generating optimal 8–10 amino acid epitopes for loading onto MHC-I molecules [12,13,14].

Rather than serving as simple processing enzymes, ERAP1 and ERAP2 act as key editors of the immunopeptidome. By selectively trimming peptide precursors, they influence not only the quantity, but also the quality of peptides presented by MHC-I molecules, thereby shaping immune recognition.

### 2.1. ERAP1 Molecular Ruler Mechanism: A Peptide Editor

ERAP1 exhibits a distinctive structural organization that enables highly controlled peptide trimming through the so-called molecular ruler mechanism [15]. The catalytic N-terminal domain recognizes the peptide amino terminus, whereas the C-terminal regulatory domain simultaneously engages the carboxy terminus. This coordinated interaction allows ERAP1 to progressively remove N-terminal residues until the peptide becomes too short to contact both domains simultaneously, at which point trimming ceases [16]. This mechanism underlies two key features of ERAP1 activity. First, substrate selection depends on the identity of the peptide C-terminal residues, leading to preferences that closely match the anchor residue requirements of many MHC-I molecules [17]. Recent structural studies identified a substrate-specificity region in the C-terminal domain, where even single amino acid substitutions can change peptide preference, highlighting how small structural differences in ERAP1 influence antigen processing [18]. Second, ERAP1 activity decreases once peptides reach the optimal length for MHC-I binding [14]. This length-sensing mechanism prevents excessive trimming and preserves antigenic epitopes. Together, these properties make ERAP1 a selective peptide editor that shapes the MHC-I immunopeptidome.

### 2.2. Functional Cooperation Between ERAP1 and ERAP2

Unlike mice and most other mammals, which express a single aminopeptidase (endoplasmic reticulum aminopeptidase associated with antigen processing, Erap1/ERAAP), humans possess two paralogous enzymes, ERAP1 and ERAP2 [19]. Although they share a common structural organization and catalytic mechanism, the two enzymes display distinct substrate preferences, indicating that ERAP2 is not simply redundant with its paralog. ERAP1 preferentially trims peptide precursors with hydrophobic N-terminal residues, including leucine, methionine, alanine, phenylalanine, and tyrosine, whereas ERAP2 preferentially recognizes substrates carrying basic N-terminal residues [12]. In addition, ERAP1 and ERAP2 can assemble into heterodimeric complexes within the ER, enhancing peptide processing efficiency compared with either enzyme alone [12,20]. However, most of the evidence supporting ERAP1-ERAP2 heterodimer formation and its functional consequences derives from in vitro biochemical studies and overexpression systems, leaving the stability and physiological relevance of this complex in vivo incompletely defined. Furthermore, the functional interplay between the two enzymes is likely to be highly context-dependent, varying according to cell type, substrate availability, and relative expression levels. Importantly, current evidence does not support a consistent model of uniform or direct functional cooperation between ERAP1 and ERAP2, suggesting that their interplay may instead be shaped by the specific cellular and immunopeptidomic context.

The activities of ERAP1 and ERAP2 are also functionally coordinated. ERAP1 preferentially processes longer peptide precursors, whereas ERAP2 is more efficient toward shorter intermediates [21]. In turn, peptides generated by ERAP2 can competitively inhibit ERAP1, while longer substrates suppress ERAP2 activity, creating a reciprocal regulatory circuit that limits over-trimming and preserves peptides suitable for MHC-I presentation [21].

### 2.3. Genetic Diversity and ERAP Function

The functional consequences of ERAP-mediated antigen processing are further shaped by extensive genetic variation at both the *ERAP1* and *ERAP2* loci. In ERAP1, multiple single nucleotide polymorphisms (SNPs) combine into common allotypes with distinct enzymatic properties, influencing substrate preference, catalytic activity, and ultimately the repertoire of peptides presented by MHC-I molecules [22,23]. These naturally occurring allotypes represent a major source of inter-individual variability in antigen presentation. In contrast, genetic variation at the *ERAP2* locus primarily regulates protein expression. The best-characterized polymorphism is a splice-site variant that generates an unstable transcript subject to nonsense-mediated decay, resulting in complete loss of ERAP2 expression in individuals homozygous for the corresponding haplotype, which is estimated to occur in approximately 25% of the population [24]. However, recent evidence indicates that ERAP2 regulation extends beyond this single polymorphism. Long-range chromatin interactions involving regulatory elements within the neighboring leucyl and cystinyl aminopeptidase (*LNPEP*) locus contribute to the control of ERAP2 expression independently of the canonical splice-site variant, revealing a more complex regulatory landscape than previously recognized [25].

Because these polymorphisms can influence basal enzymatic activity and substrate processing, they may also modulate the efficacy and pharmacodynamic response to ERAP1/ERAP2-targeted therapies. In particular, individuals carrying low-activity or null alleles may exhibit attenuated or otherwise altered responses to ERAP1 inhibition. These observations raise the possibility that ERAP1/ERAP2 genotyping could contribute to patient stratification and treatment selection in the clinical development of ERAP-targeted therapies. However, this hypothesis remains to be formally evaluated in both preclinical and clinical studies.

### 2.4. ERAP-Dependent Peptide Editing as a Determinant of Immune Recognition

The physiological importance of ERAP-mediated peptide editing is underscored by strong genetic associations with immune-mediated diseases, including ankylosing spondylitis (AS), psoriasis, Behçet’s disease, and birdshot chorioretinopathy, where altered peptide processing contributes to disease susceptibility [26,27,28,29]. ERAP SNPs have also been implicated in antiviral immunity by modulating CD8^+^ T-cell responses to pathogens such as hepatitis C virus and HIV-1 [30,31], as well as in pregnancy-associated hypertensive disorders [32].

These findings established ERAP1 and ERAP2 enzymes as major regulators of immune recognition. In cancer, where the repertoire of MHC-I-bound peptides determines the immunological visibility of malignant cells, alterations in ERAP activity influence both CD8^+^ T-cell recognition and NK-cell responses. This central role in shaping tumor immunogenicity provides the basis for understanding the broader, non-canonical functions of ERAP aminopeptidases in cancer biology.

## 3. Moonlighting Functions of ERAP Aminopeptidases: Beyond Antigen Processing

**Figure 1:**
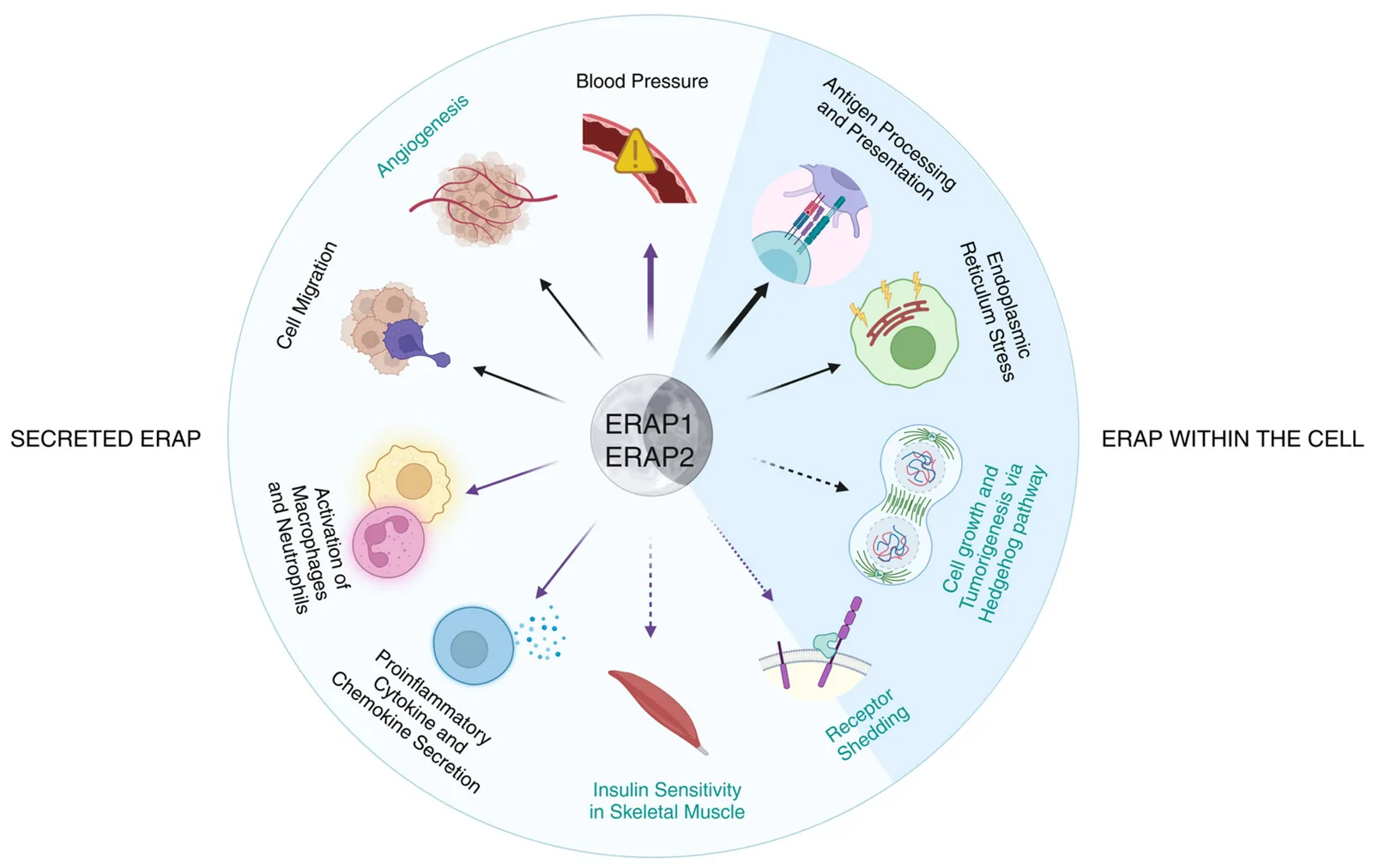
Moonlighting functions of ERAP aminopeptidases. Schematic overview of the multifunctional roles of ERAP1 and ERAP2 according to their cellular localization. The central “moon” highlights the dual nature of ERAP functions, distinguishing intracellular activities (ERAP within the cell; dark blue background) from extracellular functions mediated by secreted ERAP proteins (light blue background). Biological processes currently described exclusively for ERAP1 are indicated as green labels. Black labels indicate roles shared by both ERAP1 and ERAP2. Arrow styling reflects the level of scientific confidence supporting each mechanism: thick solid arrows represent established functions validated by extensive genetic and biochemical evidence; standard solid arrows denote emerging roles supported by reproducible data; dashed arrows indicate preliminary or proposed functions described in initial reports; and dotted arrows highlight debated or unresolved mechanisms requiring further validation. Arrow color indicates functions that have been established in cancer settings (black) and in non-cancer settings exclusively (purple). Created in BioRender.com. Fruci, D. (2026) https://BioRender.com/1piih9y (accessed on 1 September 2026).

Although ERAP1 and ERAP2 were originally identified as key components of the MHC-I APP pathway, accumulating experimental evidence suggests that their biological functions may extend beyond peptide editing (Figure 1). The concept of “moonlighting proteins” refers to proteins that perform multiple, mechanistically distinct functions often depending on cellular localization, interacting partners, or tissue context [33]. ERAP aminopeptidases exemplify this concept, with accumulating evidence implicating them in a broad range of physiological and pathological processes that extend beyond their canonical role in antigen presentation.

### 3.1. Moonlighting Functions in Cancer

The non-canonical activities of ERAP aminopeptidases have attracted growing interest in oncology, where they have been implicated in shaping multiple aspects of tumor biology independently of their effects on antigen presentation. Emerging evidence indicates that ERAP1 and ERAP2 may contribute to tumor progression by regulating angiogenesis, cell migration, intracellular signaling and stress-adaptation pathways, highlighting functions that may be therapeutically relevant.

#### 3.1.1. ERAP Enzymes in Tumor Angiogenesis and Cell Migration

Angiogenesis is a hallmark of cancer that sustains tumor growth and metastatic dissemination [34]. In addition to their intracellular enzymatic activity, ERAP proteins appear to influence vascular remodeling through mechanisms unrelated to MHC-I peptide processing. ERAP1 has been proposed to regulate endothelial responses by modulating the metabolism of bioactive peptides and extracellular signaling pathways involved in angiogenesis [35].

Evidence also implicates ERAP aminopeptidases in the regulation of cell motility. ERAP1 has been associated with endothelial and leukocyte migration [36,37], suggesting broader roles in cytoskeletal remodeling, vascular homeostasis, and vascular endothelial growth factor (VEGF)-dependent signaling. However, whether this pro-migratory function of ERAP1 extends to the cancer setting has not yet been directly demonstrated. In contrast, the role of ERAP2 in cancer motility has been investigated more extensively. In oral squamous cell carcinoma, ERAP2 expression is significantly increased compared with adjacent normal epithelium, while its silencing reduces tumor cell migration and invasion in vitro, suggesting that ERAP2 may contribute to tumor progression and metastatic behavior [38].

Although the underlying molecular mechanisms remain incompletely defined, these findings suggest that ERAP aminopeptidases may influence both tumor cells and stromal components through pathways distinct from antigen processing, expanding their biological and therapeutic significance.

#### 3.1.2. ERAP1 as a Regulator of Tumor-Intrinsic Signaling

Initial preclinical studies have revealed an unexpected role for ERAP1 in modulating oncogenic signaling pathways. In medulloblastoma (MB), ERAP1 was identified as a positive regulator of the Hedgehog (Hh) pathway, a developmental signaling cascade that drives tumor initiation and maintenance. Bufalieri and colleagues demonstrated that genetic or pharmacological inhibition of ERAP1 attenuated Hh signaling, reduced tumor cell proliferation, and improved survival in preclinical models (see Section 5.1.4) [39,40]. Importantly, these effects were observed independently of antigen presentation, suggesting that ERAP1 may contribute to tumor cell fitness through mechanisms distinct from its established role in immune surveillance. Although these findings require validation across additional tumor types, they raise the possibility that ERAP1 may exert broader tumor-promoting functions beyond its canonical role in antigen processing.

#### 3.1.3. ERAP Aminopeptidases and ER Stress

Evidence linking ERAP enzymes to ER stress has emerged primarily from studies of ERAP2. In pancreatic ductal adenocarcinoma, ERAP2 knockdown impaired the ability of pancreatic stellate cells to promote tumor cell migration and invasion by inhibiting ER stress-induced autophagy, resulting in reduced fibrosis and tumor growth in xenograft models [41]. These findings identified ER stress and downstream unfolded protein response (UPR) signaling as key mediators of ERAP2-dependent stromal activation [41]. A similar connection between ERAP1 and ER stress has also been reported in auto-inflammatory contexts. In a preclinical model of AS, ERAP1 depletion was associated with reduced UPR activation [42], suggesting that ERAP1 may also participate in cellular stress-adaptation pathways.

Although the available evidence remains limited, these findings suggest that ERAP aminopeptidases may contribute to the adaptive response to chronic ER stress, potentially promoting autophagy, stromal remodeling, and other processes that support tumor progression. Whether ERAP inhibition could synergize with pharmacological targeting of the UPR represents an intriguing, but still unexplored, therapeutic strategy.

### 3.2. Moonlighting Functions Outside Cancer

While this review focuses on the role of ERAP aminopeptidases in cancer, it is worth noting that ERAP1 and ERAP2 have been implicated in several biological processes unrelated to cancer. Beyond their roles in tumor biology, these enzymes are involved in the regulation of blood pressure, metabolic homeostasis, and innate immune activation, and have also been implicated in modulating cytokine receptor availability. Recognizing these broader physiological functions is essential when evaluating the potential on-target effects of systemic ERAP inhibition, particularly in the context of therapeutic development.

#### 3.2.1. ERAP1 and ERAP2 in the Renin-Angiotensin System and Blood Pressure Regulation

A well-established non-canonical function of ERAP1, and to a lesser extent ERAP2, is its involvement in the regulation of blood pressure and vascular tone through the renin-angiotensin system (RAS). While Angiotensin II (Ang II) normally drives vasoconstriction and inflammation, extracellular ERAP1 and ERAP2 sequentially cleave Ang II into Ang III and Ang IV. These cleavage products counteract Ang II by lowering blood pressure, reducing inflammation, and promoting nitric oxide (NO) production [43,44,45]. Polymorphic variants that reduce ERAP cleavage efficiency can therefore shift this balance toward inflammation, hypertension, and atherosclerosis. This axis became critical during SARS-CoV-2 infection, where angiotensin-converting enzyme 2 downregulation raised circulating Ang II. Our group hypothesized that individuals with impaired ERAP1/ERAP2 activity fail to compensate for this Ang II accumulation, increasing their risk of severe COVID-19 and related cardiopulmonary injury [46]. Adding further complexity, in 2022 a “short” form of ERAP2 was reported [47]. This truncated ERAP2 is secreted specifically by M2-like macrophages independent of the classical gene variants, directly binds insulin-regulated aminopeptidase/angiotensin type 4 receptor (IRAP/AT4R), and may help preserve RAS balance even when canonical ERAP function is impaired [48]. Looking ahead, given the crucial role of RAS signaling, NO production, and M2-like macrophage polarization in the tumor microenvironment (TME), exploring whether this ERAP-mediated vascular and metabolic axis also operates in the context of tumor-associated angiogenesis and stroma remodeling represents an intriguing avenue for future investigation.

#### 3.2.2. Extracellular ERAP1 and ERAP2 in Cytokine Dynamics and Innate Immune Activation

One of the first proposed non-canonical functions of ERAP1 is its involvement in the regulation of inflammatory signaling through the processing and shedding of cytokine receptors. ERAP1 has been implicated in the extracellular cleavage of membrane-associated receptors, such as the IL-1 receptor type II (IL-1R2) and Tumor Necrosis Factor (TNF) receptor family members, thereby influencing the availability of soluble receptor forms and modulating cytokine activity [49,50]. However, this role remains controversial, as it is supported by only a small number of studies from a single group and has never been independently replicated, despite the growing interest in ERAP1 biology. Consequently, the contribution of ERAP1 to cytokine receptor shedding remains unresolved and requires further experimental validation.

Beyond this debated shedding function, accumulating evidence suggests that extracellular ERAP1 and ERAP2 may directly promote cytokine secretion and modulate the activation of innate immune cells. In 2011, Goto and colleagues first described that macrophages release ERAP1 in response to interferon (IFN)-γ or bacterial stimuli. This activation pathway involves the induction of IFN-β and TNF-α, an influx of intracellular calcium, and the activation of calmodulin, which together trigger ERAP1 release. Once secreted, ERAP1 promotes macrophage phagocytosis and contributes to NO production, contributing to tumoricidal, antimicrobial, and immunomodulatory responses [51,52,53,54].

A similar regulated secretion mechanism has also been described for ERAP2. Paralleling ERAP1, Saulle et al. showed that human monocyte-derived macrophages (MDMs) release ERAP2 specifically upon activation with IFN-γ and lipopolysaccharide (LPS), whereas resting cells do not. The secreted form of ERAP2 acts beyond its canonical peptide-trimming role: it reduces human immunodeficiency virus (HIV)-1 replication in peripheral blood mononuclear cells (PBMCs) while boosting CD8^+^ T cell activation and inflammatory markers [55]. These findings provided initial evidence that ERAP2 can be released by immune cells to function as an extracellular immunostimulatory signal.

Aldhamen and colleagues extended this concept to systemic immunity [56]. They showed that extracellular ERAP1 stimulates human PBMCs to produce inflammatory cytokines and chemokines, enhancing immune cell activation. Notably, autoimmune disease-associated ERAP1 variants triggered a stronger innate immune response than the protective reference variant. This established that naturally occurring ERAP1 polymorphisms could tune extracellular innate immune signaling, offering a proposed mechanistic link between ERAP1 genetic variation and autoimmune disease risk. More recently, Saulle et al. [37] showed that recombinant ERAP1 and ERAP2 are internalized by neutrophils and drive their activation, increasing cytokine release, migration, autophagy, and phagocytosis.

Together, these preliminary findings suggest that extracellular ERAP1 and ERAP2 may contribute to the regulation of innate immune cell homeostasis and shape inflammatory responses under conditions of cellular stress. Whether these immunostimulatory properties are maintained, altered, or co-opted within the TME remains an intriguing and largely unexplored question, warranting further investigation in the context of cancer.

#### 3.2.3. ERAP1 and Insulin Resistance in Skeletal Muscle

ERAP1 has also recently been described as a potential metabolic regulator. Under pro-inflammatory conditions, such as a high-fat diet or chronic inflammation, ERAP1 can act as a hepatokine and promote insulin resistance in skeletal muscle [57]. Niu and colleagues proposed that, once released from hepatocytes into the bloodstream, circulating ERAP1 is taken up by skeletal muscle, where it binds the β2-adrenergic receptor (ADRB2). This interaction impairs downstream insulin signaling and disrupts glucose homeostasis.

While these preliminary findings suggest that circulating ERAP1 may represent a potential therapeutic target in metabolic dysregulation and type 2 diabetes, evidence for this non-canonical endocrine mechanism remains limited to early experimental models and requires independent validation. Whether systemic levels of ERAP1 are altered in acute or chronic liver pathologies and whether they reflect underlying tissue damage or metabolic stress in human cohorts represents a crucial direction for future translational research.

## 4. Current Strategies for Pharmacological Targeting of ERAP Aminopeptidases

The development of selective ERAP inhibitors has been challenging because ERAP1, ERAP2, and the related IRAP, share a high degree of sequence and structural homology, particularly within their zinc-dependent catalytic sites [58]. As a result, the earliest medicinal chemistry efforts primarily yielded broad-spectrum compounds that inhibited multiple family members, limiting their value as selective pharmacological tools. A comprehensive review by Fougiaxis and colleagues recently summarized the current landscape of ERAP inhibitors, cataloging more than 100 compounds reported to date [59]. Most first-generation inhibitors target the highly conserved catalytic zinc-binding pocket and therefore lack selectivity for individual ERAP family members. Representative examples include phosphonic and phosphinic acid derivatives, which were the first rationally designed inhibitors of ERAP [60].

Significant progress has recently been made toward selective ERAP1 inhibition. Fragment-based drug discovery approaches identified several chemical series with low-micromolar potency and high selectivity over ERAP2 and IRAP [61]. Independently, orally bioavailable ERAP1 inhibitors have demonstrated therapeutic activity in preclinical models of colorectal cancer, inflammatory arthritis, and Hh-driven MB, providing proof of concept that pharmacological ERAP1 inhibition can exert both immunological and tumor-intrinsic effects [40,62]. The most advanced program has been developed by Grey Wolf Therapeutics, whose lead compound, GRWD5769 [63], is currently being evaluated in the phase I/II EMITT-1 clinical trial (NCT06923761) (see Section 5.2).

By comparison, the development of selective ERAP2 inhibitors remains at an earlier stage. The first nanomolar-selective compounds were described in 2022 [64], and subsequent work focused on improving their therapeutic delivery, in particular, incorporating an ERAP2 inhibitor into electrospun nanofibrous scaffolds. These nanofibers enabled sustained drug release while preserving biological activity in vitro, supporting this platform as a potential strategy for treating both cancer and immune-mediated diseases [65].

Overall, the field has evolved rapidly from non-selective enzymatic probes to clinically evaluated ERAP1 inhibitors with favorable drug-like properties. However, the structural similarity shared by ERAP1, ERAP2, and the related aminopeptidase IRAP raises concerns regarding potential off-target effects. Inhibition of IRAP could interfere with its immune-related functions, while unintended modulation of ERAP1 or ERAP2 may perturb their reciprocal functional interplay and, consequently, alter the composition of the immunopeptidome in unpredictable ways. These considerations underscore the need to develop highly selective inhibitors with sufficient specificity to discriminate among these closely related paralogs. Although ERAP2-selective compounds are still in preclinical development, advances in isoform-selective chemistry and targeted drug delivery are expected to accelerate their translation toward therapeutic applications.

## 5. Therapeutic Implications of ERAP Targeting in Cancer

**Figure 2:**
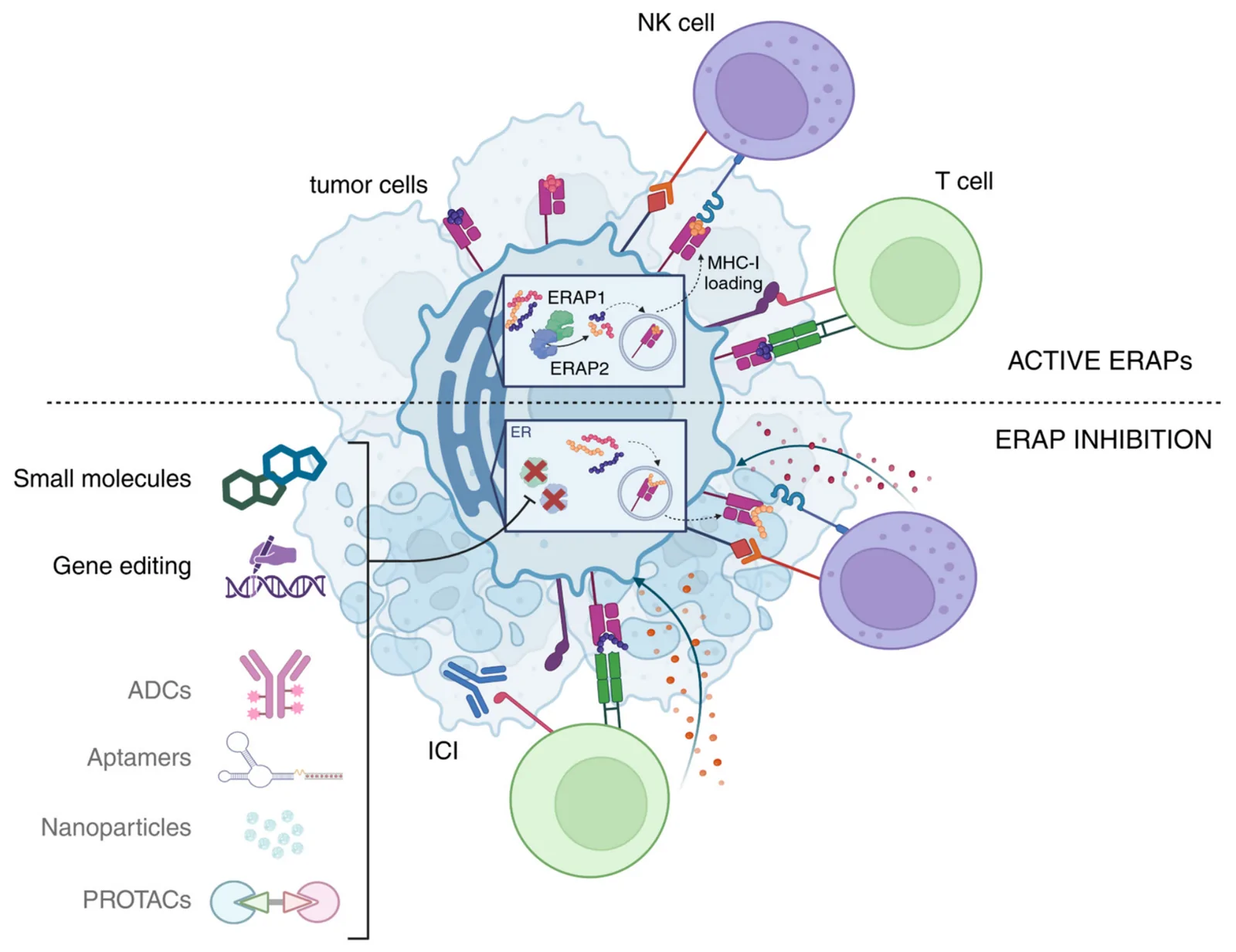
Therapeutic targeting of ERAP1 and ERAP2 to modulate tumor antigen presentation and antitumor immunity. Functionally active ERAP1 and ERAP2 (top panel, above the dashed line) trim peptide precursors in the endoplasmic reticulum (ER), shaping the major histocompatibility complex class I (MHC-I) immunopeptidome and influencing T-cell and NK-cell recognition. Dashed arrows depict the MHC-I peptide loading. Pharmacological or genetic inhibition of ERAP activity (bottom panel, below the dashed line) can remodel peptide processing and MHC-I presentation, modulating the immunopeptidome and potentially altering immune recognition and promoting cytotoxic responses (blue arrows). ERAP1/ERAP2 can be targeted through small-molecule inhibitors and gene-editing approaches (black T-bar), while emerging strategies (greyed-out) like antibody–drug conjugates (ADCs), aptamers, nanoparticles, and proteolysis-targeting chimeras (PROTACs); represent potential strategies for future ERAP-targeted therapies. ERAP inhibition in cancer may represent a promising immunomodulatory strategy, particularly in combination with immune-checkpoint inhibitors (ICIs), although its effects are context-dependent and influenced by tumor type, ERAP genotype and expression, and the tumor immunopeptidome. Created in BioRender.com. Fruci, D. (2026) https://BioRender.com/3y3k86i (accessed on 1 September 2026).

The therapeutic potential of ERAP1 and ERAP2 inhibition arises from their ability to remodel the repertoire of peptides displayed by tumor cells, thereby altering their interaction with both adaptive and innate immune effectors (Figure 2). Unlike conventional immunotherapies, which primarily amplify immune-cell activation, ERAP inhibition acts upstream by modifying tumor antigenicity itself. This distinctive mechanism provides opportunities not only to enhance immune recognition, but also to overcome resistance in poorly immunogenic tumors. Beyond its effects on antigen presentation, accumulating evidence indicates that ERAP enzymes also regulate NK-cell inhibitory pathways and tumor-intrinsic signaling networks (Table 1). Consequently, pharmacological targeting of ERAP may simultaneously influence immune surveillance and cancer cell biology, providing a strong rationale for its therapeutic development.

### 5.1. Preclinical Studies

#### 5.1.1. ERAP1 Inhibition Enhances Tumor Immune Recognition

ERAP1 inhibition limits excessive peptide trimming, allowing the presentation of cryptic or alternative epitopes that are normally destroyed during antigen processing [14]. The resulting diversification of the tumor peptide repertoire creates new targets for cytotoxic lymphocytes and may be particularly beneficial in tumors characterized by low immunogenicity and poor responsiveness to ICIs.

The first evidence that ERAP1 loss could enhance antitumor immunity came from murine lymphoma models, where ERAAP deficiency altered the repertoire of MHC-I-bound peptides and promoted efficient immune-mediated tumor rejection [66]. Although both CD8^+^ and CD4^+^ T cells contributed to tumor control, NK cells were responsible for the early phase of tumor elimination [66]. Mechanistically, ERAAP deficiency did not significantly affect MHC-I surface expression but instead modified the quality of peptide-MHC-I complexes. This phenomenon impaired engagement of inhibitory NK-cell receptors and increased susceptibility to NK-cell-mediated killing, demonstrating that ERAP1 regulates immune recognition primarily by controlling peptide composition rather than MHC-I abundance [66]. Together, these studies established proof-of-principle that manipulating ERAP1 can increase tumor immunogenicity and promote immune-mediated tumor elimination.

#### 5.1.2. Remodeling of the Tumor Immunopeptidome

Subsequent studies extended these observations across multiple tumor models, including melanoma, colorectal cancer, lymphoma, and neuroblastoma (NB). Both pharmacological inhibition and genetic depletion of ERAP1 and ERAP2 broadened the repertoire of MHC-I ligands, increased the presentation of previously cryptic peptides, delayed tumor growth, and improved responsiveness to immunotherapy [67,68,69,70,71,72]. Although pharmacological inhibition and genetic ablation do not necessarily produce identical outcomes, likely reflecting the distinct consequences of complete and sustained ERAP1 loss versus partial or transient inhibition of its catalytic activity, both approaches ultimately converge on a common effect: increased diversification of the immunopeptidome. These findings support the concept of tumor immunopeptidome engineering, whereby selective modulation of ERAP enzymes can generate antigenic landscapes that are more efficiently recognized by the immune system. This strategy may be particularly attractive in immunologically “cold” tumors, where ERAP inhibition could enhance the presentation of previously underrepresented epitopes and thereby partially overcome the limited neoantigen repertoire associated with a low tumor mutational burden. In NB, ERAP1 inhibition combined with the histone deacetylase inhibitor entinostat increased MHC-I expression, diversified the tumor immunopeptidome, enhanced lymphocyte recognition, and restored sensitivity to ICIs [69].

#### 5.1.3. Modulation of NK-Cell Checkpoints

In addition to its effects on adaptive immunity, ERAP1 regulates NK-cell activity by controlling the peptide repertoire presented by both classical and non-classical MHC-I molecules. Because inhibitory NK receptors recognize peptide-MHC-I complexes rather than MHC-I molecules alone, even subtle alterations in peptide sequence can profoundly modify receptor engagement [10]. Accordingly, ERAP1 deficiency reduces inhibitory signaling through killer-inhibitory receptors (KIRs) and CD94/NKG2A, resulting in enhanced NK-cell cytotoxicity [73,74]. More recently, ERAP1 was shown to regulate the HLA-E/NKG2A immune checkpoint by controlling the generation of HLA-E-binding peptides required for receptor activation [75]. Interestingly, species-specific differences have emerged: whereas ERAP1 deletion alone disrupts this pathway in mice, simultaneous loss of both ERAP1 and ERAP2 is required in human cells.

The contribution of ERAP2 to NK-cell regulation remains less well defined. Further studies are therefore needed to elucidate its specific role, particularly whether ERAP2 influences NK-cell activity through the direct modulation of NK-cell receptor ligands or indirectly by reshaping the broader peptide repertoire available for immune recognition. Collectively, these findings indicate that ERAP inhibition can potentiate both adaptive and innate immunity, providing a compelling rationale for combination strategies with ICIs, adoptive cell therapies, and therapeutic cancer vaccines.

#### 5.1.4. Effects on Tumor-Intrinsic Pathways

The therapeutic value of ERAP1 inhibition is not exclusively immune-mediated. As discussed in Section 3, ERAP1 also regulates oncogenic signaling independently of antigen presentation. In MB, pharmacological inhibition or genetic depletion of ERAP1 impaired Hh signaling, reduced tumor cell proliferation, and prolonged survival in preclinical models [39,40]. These observations suggest that ERAP1-targeted therapies may exert complementary mechanisms of action by simultaneously enhancing tumor immunogenicity and directly suppressing signaling pathways that sustain tumor growth.

### 5.2. Clinical Studies

The clinical translation of ERAP1 targeting has recently begun following extensive preclinical validation. The most advanced compound, GRWD5769 (Grey Wolf Therapeutics), is an orally bioavailable, selective ERAP1 inhibitor currently being evaluated in the phase I/II EMITT-1 clinical trial (NCT06923761) in combination with the anti-programmed cell death protein 1 (PD-1) antibody cemiplimab in patients with advanced solid tumors previously treated with ICIs. Early results presented at ASCO 2024 and ASCO 2026 indicate that GRWD5769 is well tolerated, with no unexpected toxicities beyond those typically associated with ICI therapy [76,77]. At the interim analysis, all six phase Ib expansion cohorts had been fully recruited (*n* = 81), including patients with non-small cell lung cancer (*n* = 14), urothelial carcinoma (*n* = 14), hepatocellular carcinoma (*n* = 15), cervical cancer (*n* = 15) and squamous cell carcinoma of the head and neck (*n* = 11) with secondary resistance following first line anti-PD-1 therapy, as well as patients with microsatellite-stable colorectal cancer (MSS-CRC, *n* = 12) without liver metastases. After a median follow up of 6.0 months, durable responses were observed across all cohorts, with objective response rate (ORR) ranging from 10 to 33% and durable clinical benefit (DCB) rates from 26 to 57%. Median progression-free survival (PFS) ranged from 1.9 to 7.5 months across evaluable cohorts.

Translational analyses provided further support for the proposed mechanism of ERAP1 inhibition. Patients achieving clinical benefit exhibited a marked increase in T-cell receptor (TCR) repertoire diversity, driven by the expansion of low-frequency, putative de novo TCR clonotypes. Responders also showed cyclical changes in Vβ gene usage, consistent with broad T-cell clonal expansion and contraction and suggesting dynamic changes in antigen recognition following ERAP1 inhibition. These findings suggest a dual immunomodulatory mechanism whereby GRWD5769, in combination with anti-PD-1, both reprograms antigen-experienced T-cell responses and promotes the emergence of de novo antitumor T-cell clonotypes. This activity is consistent with the ability of ERAP1 inhibition to remodel the tumor immunopeptidome and broaden the repertoire of tumor-associated antigens available for immune recognition.

**Table 1:** Summary of the evidence supporting the roles of ERAP1 and ERAP2 in cancer.

Cancer Type	Target Enzyme	Type of Inhibition	Combination Therapy	Context	Main Findings	Year of Publication	Reference
Solid malignancies	ERAP1	Inhibitor (GRWD5769)	Cemiplimab (anti-PD-1)	Clinical trial phase I/II	Objective response, durable disease control, low toxicity	- *	EMITT-1 trial, NCT06923761
Murine colorectal cancer (CT26)	ERAP1	Inhibitor (GSK235)	None	In vitro, in vivo	Modulation of cancer cell immunopeptidome, tumor growth control	2026	[62]
Murine hedgehog-dependent medulloblastoma	ERAP1	Inhibitor (canthin-6-one, N1)	None	In vitro, in vivo	In vitro and in vivo growth control, improved host survival	2026	[40]
Human melanoma (A375)	ERAP1	Genetic inhibition (CRISPR-Cas9), inhibitor (Compound 3)	None	In vitro	Modulation of cancer cell immunopeptidome, proteome, metabolism and stress response	2025	[71]
Murine and human cell lines	ERAP1, ERAP2	Genetic inhibition (CRISPR-Cas9)	Anti-PD-1	In vitro, in vivo	Alteration of HLA-E peptidome, inactivation of NKG2A-HLA-E checkpoint, sensitization to immunotherapy (mouse tumor models)	2024	[75]
Murine neuroblastoma (9464D)	ERAP1	Genetic inhibition (CRISPR-Cas9)	Entinostat, anti-PD-1	In vitro, in vivo	Enhanced immune-mediated killing, immunopeptidome modulation, sensitization to immunotherapy	2024	[69]
Human melanoma (A375)	ERAP1	Genetic inhibition (CRISPR-Cas9), inhibitor (Compound 1)	None	In vitro	Modulation of cancer cell immunopeptidome	2023	[72]
Human leukemia (MOLT-4)	ERAP2	Inhibitor (DG011A)	None	In vitro	Modulation of cancer cell immunopeptidome	2022	[70]
Murine hedgehog-dependent medulloblastoma	ERAP1	Genetic inhibition (shRNA), inhibitor (Leu-SH)	None	In vitro, in vivo	ERAP1 positively regulates the hedgehog pathway, in vitro and in vivo modulation of tumor cell growth	2019	[39]
Human melanoma (A375)	ERAP1	Inhibitor (DG013A)	None	In vitro	Modulation of cancer cell immunopeptidome	2019	[68]
Human cell lines	ERAP1	Genetic inhibition (shRNA), inhibitor (Leu-SH)	None	In vitro	NK activation by disrupting inhibitory KIR and CD94-NKG2A recognition of peptide–MHC-I complexes	2015	[73]
Murine lymphoma cell lines	ERAP1	Genetic inhibition (miRNA)	None	In vitro, in vivo	MHC-I peptide-loading defects, NK-driven tumor rejection	2011	[66]

* Preliminary results from an early-phase combination trial of GRWD5769 with cemiplimab presented at ASCO conferences, not yet published in peer-reviewed journals.

Overall, the clinical activity observed in heavily pretreated patients with secondary anti-PD-1 resistance, as well as in MSS-CRC, provides preliminary evidence that GRWD5769 may help overcome resistance to PD-1 blockade. Stage 2 cohort expansions are currently ongoing to further evaluate the efficacy and safety of the combination therapy and inform the design of a randomized phase II study.

Beyond adaptive immunity, the therapeutic effects of ERAP1 inhibition may also involve innate immune mechanisms, as alterations in peptide presentation could simultaneously enhance CD8^+^ T-cell responses and modulate inhibitory signaling in NK cells.

Future clinical studies will be essential to identify the patients most likely to benefit from ERAP1-targeted therapies and to establish robust predictive biomarkers. Candidate biomarkers include human leukocyte antigen class I (HLA-I) genotype, ERAP1 allotypes, baseline immunopeptidome composition, patterns of immune-cell infiltration, and molecular features associated with defects in APP. Integrating these parameters with pharmacodynamic and immune-response profiling may ultimately enable more precise patient selection and guide the optimal combination of ERAP1 inhibitors with immune checkpoint blockade.

### 5.3. Potential Strategies for ERAP-Targeted Therapy

Because ERAP aminopeptidases contribute to physiological immune homeostasis, prolonged systemic inhibition may produce unintended effects beyond the tumor compartment. Future therapeutic approaches should therefore aim to achieve selective and, ideally, tumor-restricted modulation of ERAP activity while preserving its physiological functions in normal tissues. To date, no targeted delivery strategy has been specifically developed for ERAP-directed therapeutics. Nevertheless, several emerging therapeutic platforms may provide a conceptual framework for achieving localized ERAP modulation in cancer.

One potential approach is the use of antibody–drug conjugates (ADCs) directed against tumor-associated antigens to selectively deliver ERAP inhibitors or ERAP-modulating agents to malignant cells. By exploiting the specificity of antibody-mediated recognition, ADCs could, in principle, restrict pharmacological activity to tumor tissues while limiting systemic exposure [78]. Engineered nucleic acid aptamers represent another potentially attractive strategy, owing to their high target affinity, low immunogenicity, and structural versatility for conjugation with therapeutic cargos. These properties could be exploited to achieve selective delivery of ERAP1- or ERAP2-modulating agents to specific tumor types or cellular populations [79]. Extracellular vesicles and engineered nanoparticles offer additional opportunities for localized delivery. Their capacity to transport proteins, nucleic acids, and small molecules across cellular compartments makes them particularly appealing as vehicles for ERAP inhibitors, gene-silencing molecules, or other regulatory cargos. By facilitating transient and spatially restricted modulation of ERAP activity within the TME, these platforms could potentially enhance therapeutic efficacy while limiting systemic exposure and associated immune-related toxicity [80,81]. A distinct and potentially more transformative approach is represented by targeted protein degradation technologies, including proteolysis-targeting chimeras (PROTACs). Unlike conventional inhibitors, which transiently suppress enzymatic activity, PROTACs promote the selective degradation of their target proteins and could therefore achieve more sustained depletion of ERAP1 or ERAP2 within tumor cells [82]. This strategy may be particularly relevant in tumors in which ERAP enzymes exert both antigen-processing and tumor-intrinsic functions, as protein degradation could simultaneously interfere with immune-evasion mechanisms and oncogenic pathways.

Although these approaches remain largely conceptual in the context of ERAP-targeted therapy, they provide a framework for overcoming one of the major challenges associated with systemic ERAP inhibition, namely achieving sufficient antitumor activity while minimizing disruption of physiological immune functions. Their translation into clinically viable strategies will require extensive optimization and validation in appropriate preclinical models, including assessment of tumor selectivity, pharmacokinetics, tissue distribution, and potential toxicity. Nevertheless, the development of tumor-directed delivery and protein-degradation approaches could ultimately expand the therapeutic window of ERAP-targeted interventions and facilitate their integration into precision cancer immunotherapy.

## 6. Conclusions and Future Perspectives

ERAP aminopeptidases have emerged as multifunctional regulators of cancer biology whose activities extend well beyond their canonical role in MHC-I antigen processing.

While their ability to edit the tumor immunopeptidome remains the primary mechanism underlying immune recognition, accumulating evidence indicates that ERAP1 and ERAP2 also participate in angiogenesis, cell migration, ER stress responses, inflammatory signaling, and tumor-intrinsic oncogenic pathways. These moonlighting functions position ERAP enzymes at the intersection of tumor cell biology and antitumor immunity, broadening their relevance as therapeutic targets.

Among the two family members, ERAP1 is currently the most advanced therapeutic target. Extensive preclinical studies have demonstrated that its inhibition reshapes the repertoire of tumor-associated peptides, enhances both T-cell- and NK-cell-mediated immune responses, and improves the efficacy of immunotherapeutic approaches. More recent findings further indicate that ERAP1 inhibition may directly interfere with oncogenic signaling pathways, suggesting that its antitumor activity is likely to result from the convergence of immune-dependent and tumor-intrinsic mechanisms. The ongoing clinical evaluation of selective ERAP1 inhibitors will determine whether these encouraging preclinical observations translate into meaningful clinical benefits.

Despite these advances, several important challenges remain. Although current pharmacological approaches primarily rely on small-molecule enzymatic inhibitors, achieving tumor-selective ERAP1 modulation may ultimately require integration of advances in medicinal chemistry with innovative therapeutic platforms, including ADCs, aptamer-based delivery systems, and targeted protein degraders such as PROTACs.

The integration of selective ERAP-targeting approaches with existing immunotherapies represents a promising avenue for future cancer treatment. By increasing the diversity of tumor-associated antigens, ERAP modulation has the potential to enhance the efficacy of ICIs, adoptive T-cell and NK-cell-based therapies, and therapeutic cancer vaccines, particularly in poorly immunogenic tumors. Realizing this potential, however, will require a better understanding of how ERAP1 and ERAP2 genetic variants, HLA background, and tumor immunopeptidome composition shape therapeutic responses. These molecular determinants, together with immune infiltration patterns, are likely to serve as predictive biomarkers for patient stratification and should be integrated into future translational and clinical studies.

The therapeutic potential of ERAP modulation extends beyond restoring antigen presentation. By simultaneously reshaping the tumor immunopeptidome and influencing tumor-intrinsic biological processes, selective targeting of ERAP enzymes offers the opportunity to enhance immune recognition while disrupting complementary pathways that support tumor progression. As our understanding of both the canonical and moonlighting functions of ERAP aminopeptidases continues to expand, these enzymes are emerging as attractive targets for next-generation precision cancer immunotherapy, linking antigen processing, tumor biology, and personalized therapeutic intervention within a unified framework.

Nevertheless, several important limitations should be considered when interpreting the current evidence on ERAP-targeted cancer immunotherapy. Most mechanistic and functional insights into ERAP1 and ERAP2 derive from preclinical and in vitro models, and it remains uncertain to what extent these systems faithfully recapitulate the complexity of the human tumor immunopeptidome and the non-canonical activities of these enzymes in vivo. Another important limitation is the unequal characterization of the two paralogs. While ERAP1 has entered clinical evaluation, the contribution of ERAP2 to tumor immunity and its functional interplay with ERAP1 remain comparatively understudied. Moreover, the complete absence of functional ERAP2 in a substantial proportion of individuals, together with the extensive genetic and functional diversity of ERAP1 allotypes, complicates the identification of patients most likely to benefit from ERAP-targeted therapies. These sources of interindividual variability underscore the need for robust predictive biomarkers capable of integrating ERAP genotype, expression, enzymatic activity, and immunopeptidomic features. Finally, the current clinical evidence for ERAP1 inhibition is derived from combination therapy with immune checkpoint blockade, making it difficult to disentangle the specific contribution of ERAP1 modulation from that of the accompanying ICI. Future studies should therefore incorporate appropriate comparator arms and comprehensive pharmacodynamic and immunological assessments to define the independent and synergistic effects of ERAP inhibition. Addressing these knowledge gaps through integrated genomic, immunopeptidomic, and biomarker-driven studies, together with rigorously designed clinical trials, will be essential for identifying responsive patient populations and establishing the optimal therapeutic context for ERAP targeting. Such efforts will ultimately determine whether ERAP-directed interventions can be rationally incorporated into precision immuno-oncology.

## Data Availability

No new data were created or analyzed in this study. Data sharing is not applicable to this article.

## Abbreviations

The following abbreviations are used in this manuscript:

ICI	Immune Checkpoint Inhibitor
NK	Natural Killer
MHC-I	Major Histocompatibility Complex class I
APP	Antigen Processing and Presentation
ER	Endoplasmic Reticulum
ERAP1	Endoplasmic Reticulum Aminopeptidase 1
ERAP2	Endoplasmic Reticulum Aminopeptidase 2
TAP	Transporter associated with Antigen Processing
ERAAP	Endoplasmic Reticulum Aminopeptidase Associated with Antigen Processing
SNP	Single Nucleotide Polymorphism
AS	Ankylosing Spondylitis
LNPEP	Leucyl and Cystinyl Aminopeptidase
VEGF	Vascular Endothelial Growth Factor
MB	Medulloblastoma
Hh	Hedgehog
UPR	Unfolded Protein Response
RAS	Renin-Angiotensin System
Ang II	Angiotensin II
NO	Nitric Oxide
IRAP	Insulin-Regulated Aminopeptidase
AT4R	Angiotensin Type 4 Receptor
IL-1R2	IL-1 Receptor type II
TME	Tumor Microenvironment
TNF	Tumor Necrosis Factor
IFN	Interferon
MDM	Monocyte-Derived Macrophage
LPS	Lipopolysaccharide
HIV	Human Immunodeficiency Virus
PBMC	Peripheral Blood Mononuclear Cell
ADRB2	β2-Adrenergic Receptor
NB	Neuroblastoma
KIR	Killer Inhibitory Receptor
PD-1	Programmed cell Death protein 1
MSS-CRC	Microsatellite-stable colorectal cancer
ORR	Objective Response Rate
DCB	Durable Clinical Benefit
PFS	Progression-Free Survival
TCR	T-cell Receptor
HLA-I	Human Leukocyte Antigen class I
ADC	Antibody–Drug Conjugate
PROTAC	Proteolysis-Targeting Chimera
